# Percutaneous large-bore mechanical thrombectomy for macroscopic fat pulmonary embolism: a case report

**DOI:** 10.1186/s42155-025-00521-6

**Published:** 2025-02-06

**Authors:** James M. Chan, Zeyad Aljeboori, Angajendra Ghosh, Benjamin Peake, Moira N. Rush, Alexandra Du Guesclin, Hui Yin Lim, Miranda Siemienowicz, Hong Kuan Kok, Goran Mitreski

**Affiliations:** 1 University of Melbourne, Melbourne, Australia; 2 Department of Intensive Care, Northern Hospital, Melbourne, Australia; 3 Department of Critical Care, University of Melbourne, Melbourne, Australia; 4 Department of Anaesthesia and Perioperative Medicine, Northern Health, Melbourne, Australia; 5 Northern Pathology Victoria, Northern Health, Melbourne, Australia; 6 Department of Haematology, Northern Health, Melbourne, Australia; 7 Department of Medicine, Northern Health, University of Melbourne, Melbourne, Australia; 8 Northern Clinical Diagnostics and Thrombovascular Research (NECTAR), Northern Health, Melbourne, Australia; 9 Interventional Radiology Service, Northern Health, Northern Imaging Victoria, Melbourne, Australia; 10 Department of Radiology, University of Melbourne, Melbourne, Australia

**Keywords:** Pulmonary embolism, Pulmonary fat embolism, CT pulmonary angiogram, Thrombectomy, Mechanical thrombectomy

## Abstract

**Background:**

Macroscopic fat pulmonary embolism is extremely uncommon. Most cases occur in the context of fat grafting or long bone fractures. Macroscopic fat pulmonary embolism may be associated with cardiopulmonary compromise and is associated with high mortality. Mechanical thrombectomy is an emerging technique in interventional radiology, primarily investigated as a therapeutic approach for thrombotic pulmonary embolism.

**Case presentation:**

We present a case report of a 73-year-old woman with macroscopic fat pulmonary embolism after a neck of femur fracture. Initially, she had severe circulatory shock, requiring multiple vasopressors and admission to the Intensive Care Unit. A percutaneous large-bore mechanical thrombectomy was performed, after which notable improvements to haemodynamic function and overall clinical trajectory were observed.

**Conclusions:**

To our knowledge, this is the first report of mechanical thrombectomy in macroscopic fat pulmonary embolism. Further research is required to better delineate the role of mechanical thrombectomy in this rare condition.

## Background

Fat embolism refers to the presence of fat in the circulation. Fat embolism may lead to fat embolism syndrome, a life-threatening condition characterised by cardiac, respiratory and neurological dysfunction [1]. Most cases of fat embolism involve microscopic fat deposits [2]. Macroscopic fat pulmonary embolism is extremely rare [2]. The few cases reported in the literature have occurred in the setting of liposuction/fat grafting [3, 4] and orthopaedic injury [2, 5, 6]. Diagnosis occurs through the identification of filling defects with fatty attenuation in the pulmonary vasculature on CT pulmonary angiography. The treatment for macroscopic fat pulmonary embolism is not well established, and is based primarily on general cardiorespiratory support [7]. In cases associated with acute cardiopulmonary collapse, extracorporeal membrane oxygenation (ECMO) may be considered [7].

Percutaneous mechanical thrombectomy refers to the manual aspiration of an embolus through a large-lumen catheter advanced endovascularly. Percutaneous mechanical thrombectomy may be an effective treatment option for thrombotic pulmonary embolism, but its use in fat pulmonary embolism has not been previously described in the literature [8, 9]. Here, we present a case of macroscopic fat pulmonary embolism after a neck of femur fracture treated with the percutaneous large-bore mechanical thrombectomy.

## Case presentation

A 73-year-old female patient sustained a displaced right femoral neck fracture after a fall. Her comorbidities included type 2 diabetes mellitus, hypertension, dyslipidaemia and osteoporosis. She had no significant preexisting cardiorespiratory disease. A right hip hemiarthroplasty was performed 36 h after initial presentation. Intraoperatively, on relocation of the hip joint, a sudden and profound drop in blood pressure was observed. A 12-lead electrocardiogram showed sinus tachycardia and signs of right ventricular strain, including right bundle branch block, T wave inversion in V1–V3, ST segment changes and the S_I_Q_III_T_III_ pattern. Intravenous fluid resuscitation was commenced, and a noradrenaline infusion was started after the insertion of a right internal jugular central venous catheter. Postoperatively, she remained intubated and was transferred to the Intensive Care Unit.

**Fig. 1:**
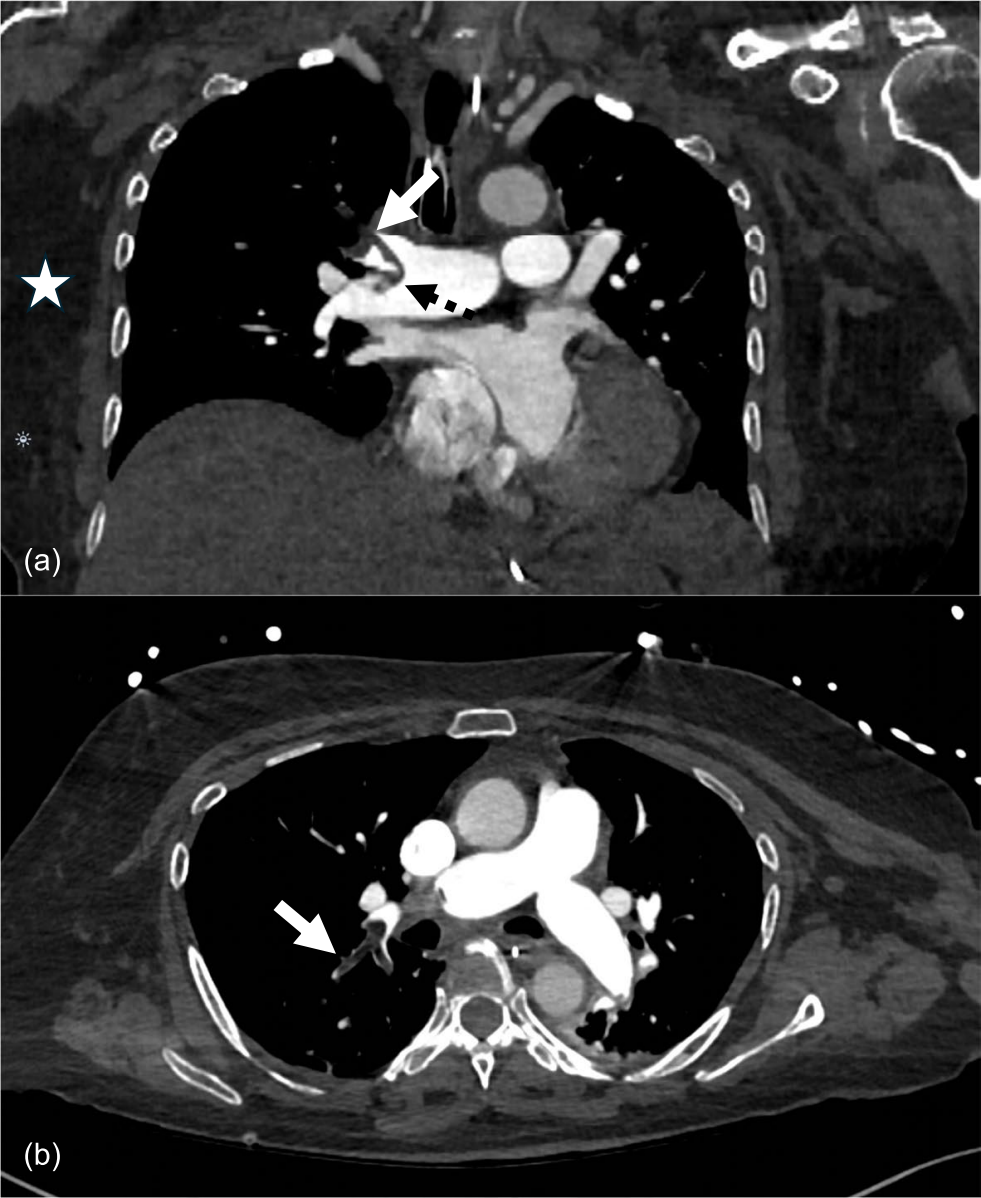
CT pulmonary angiogram. **a** Coronal soft tissue window demonstrating straddling pulmonary embolism within the right lobar and proximal segmental upper lobe branch (black dotted arrow). The filling defect in the right upper lobe lobar artery (white arrow) is of similar density to chest wall subcutaneous fat (white star). **b** Axial soft tissue window demonstrating the heterogeneous appearance of the embolus, suggesting admixed bland thrombus (white arrow)

A CT pulmonary angiogram showed multiple pulmonary emboli involving the distal right main pulmonary artery and right upper lobe proximal segmental branches (Fig. 1). The density values of these emboli were between −80 and −100 Hounsfield units, compatible with macroscopic fat. There was imaging evidence of right heart strain with bowing of the interventricular septum and an enlarged pulmonary arterial trunk. The calculated RV/LV ratio was 3.39. A transthoracic echocardiogram demonstrated multiple features of right heart dysfunction, including a dilated right ventricle with severely impaired contraction, McConnell’s sign, and a D-shaped left ventricle. Her cardiac troponin I was elevated, peaking at 7863 ng/L. This was measured with the Abbott high sensitivity assay, with a reference range of < 16 ng/L, based on the gender-specific 99th percentile for women.

**Fig. 2:**
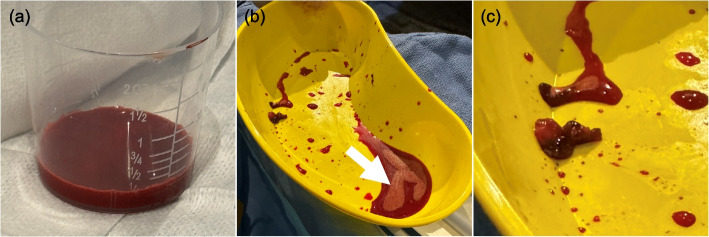
Macroscopic images of blood and tissue evacuated during large-bore mechanical thrombectomy. (**a**) Evacuated blood showing layering micelles and lipid droplets in the uppermost layer following filtration with the FlowSaver blood return system (Inari Medical, Irvine, California). (**b**) Top layer of evacuated blood with better presentation of the fatty layer (white arrow). (**c**) A macroscopic piece of fat which was aspirated and sent for histological assessment

**Fig. 3:**
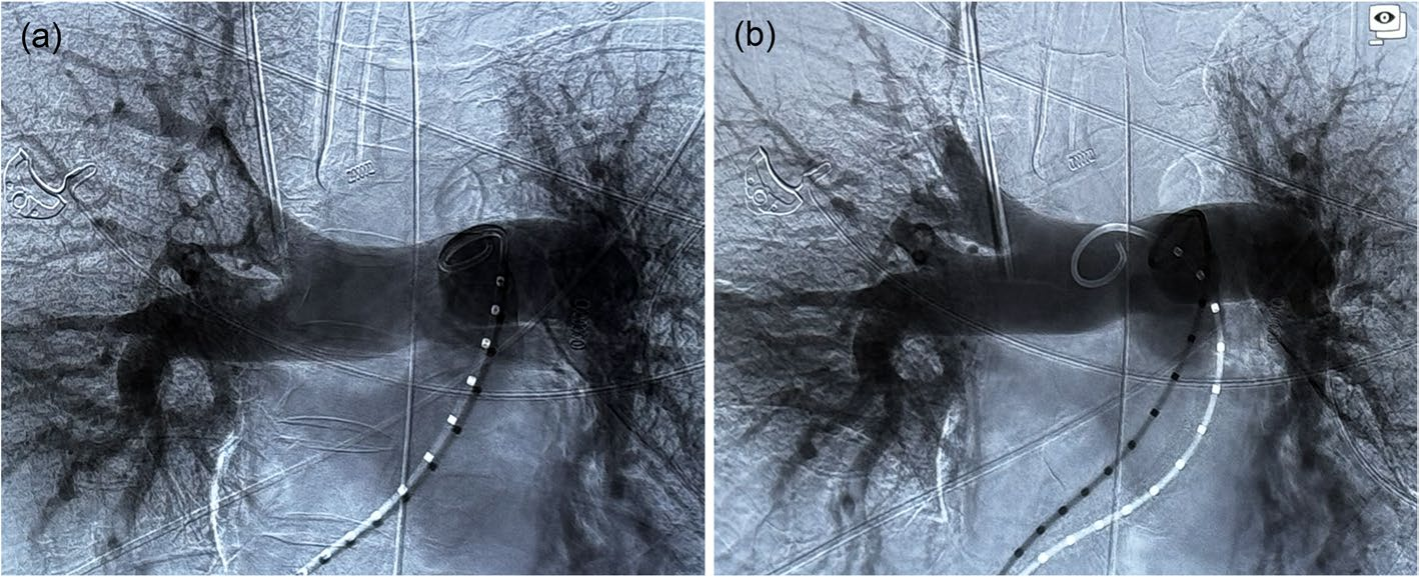
Pulmonary angiograms taken before and after large-bore mechanical thrombectomy (**a**) Pre-thrombectomy angiogram confirming right main and right upper lobe filling defects corresponding to CT pulmonary angiogram findings. (**b**) Post-thrombectomy angiogram demonstrating resolution of central emboli with residual small volume subsegmental right upper lobe emboli

**Fig. 4:**
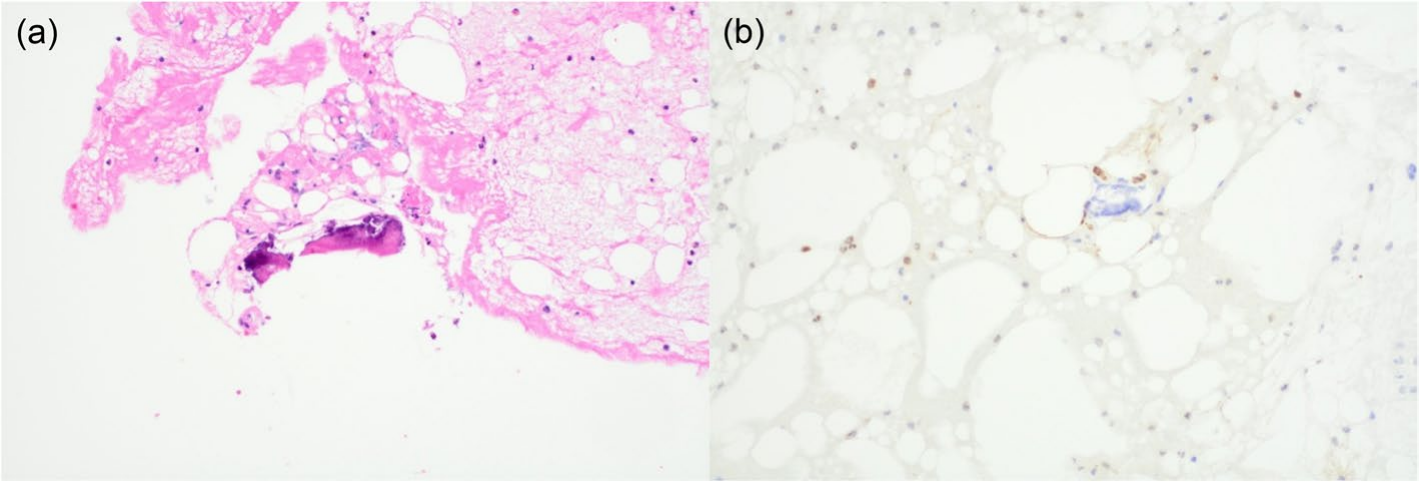
Histology of blood and tissue evacuated during large-bore mechanical thrombectomy. **a** Microscopy of retrieved tissue with haematoxylin and eosin staining showing possible adipocytes and fibrin calcification. **b** S100 immunohistochemistry showing focal nuclear staining of possible adipocytes adjacent to calcification

She had escalating vasopressor requirements, at one point needing noradrenaline 40 microg/min, argipressin, and milrinone. Considering this, she was discussed with the state-wide ECMO service. Due to her age, frailty, evidence of multi-organ failure (transaminitis and renal failure) and limited evidence supporting ECMO in fat embolism, it was decided not to commence ECMO. After extensive multidisciplinary discussion, a mechanical large-bore pulmonary thrombectomy was performed with the aim of reducing the embolic burden and alleviating the acute right heart failure. The procedure was performed in the Interventional Radiology angiography suite via right common femoral venous access. A 24 Fr Inari Intri introducer sheath (Inari Medical, Irvine, California) was inserted, followed by four thrombectomy aspirations in the right main and right upper lobe pulmonary arteries with a 24 Fr Inari FlowTriever. Although only a small amount of soft tissue was retrieved, a layer of fat was seen in the aspirated blood, suggesting the emboli were fragmented by the suction effect and filtration through the Inari FlowSaver blood return system (Fig. 2). A post-thrombectomy pulmonary angiogram showed clearance of the central emboli, with residual subsegmental emboli too distal for retrieval (Fig. 3). Immediately post procedure, her pulmonary pressure dropped from 50/13 mmHg to 41/16 mmHg. Over the ensuing days, she was weaned off vasopressor support and extubated. Histological assessment of the samples demonstrated thrombus with focal calcification and ischaemic focal adipocytes (Fig. 4). Given the finding of mixed thrombotic and fatty embolus, therapeutic enoxaparin was commenced.

The patient had a prolonged admission, complicated by ischaemic hepatitis, oliguric acute kidney injury requiring five days of continuous renal replacement therapy, and ventilator-associated pneumonia. Her acute medical issues improved over three weeks, and subsequently she was transferred to a subacute hospital for rehabilitation. After two months of rehabilitation, she was discharged home. A follow up ventilation-perfusion scan showed residual changes of pulmonary embolism. At the time this article was submitted, the patient remained under ongoing review at the haematology clinic.

## Conclusions

Recent clinical trials on percutaneous mechanical thrombectomy in intermediate-risk and high-risk pulmonary thromboembolism show promising results, including significant reductions in RV/LV ratio and pulmonary artery pressure [8–10]. A major advantage of thrombectomy over systemic thrombolysis is the lower rate of major haemorrhage and intracranial bleeding, which can lead to death or severe disability [8]. However, mechanical thrombectomy for fat pulmonary embolism has not been previously reported. In the case of macroscopic fat pulmonary embolism presented here, mechanical thrombectomy led to the rapid resolution of haemodynamic instability and notable improvement in the patient’s overall clinical trajectory. Furthermore, it allowed for histological assessment of the embolus to guide clinical decision-making, particularly around the use of anticoagulation. Our report also underscores the fat embolus fragmentation due to the suction effect and filtration through the blood return system, an important technical consideration for proceduralists.

This case highlights the utility of large-bore mechanical thrombectomy in the treatment of macroscopic fat pulmonary embolism, where treatment options are often very limited. In such cases, mechanical thrombectomy may be an effective method to rapidly reduce embolic burden and address any associated circulatory dysfunction.

## Data Availability

Not applicable.

## Abbreviation

ECMO: Extracorporeal membrane oxygenation
